# Trauma Focused Cognitive Behavioral Therapy with Children of Incarcerated Parents

**DOI:** 10.1007/s10615-017-0642-5

**Published:** 2017-10-24

**Authors:** Anna Morgan-Mullane

**Affiliations:** New York University Silver School of Social Work, 444 13th Street #2, Brooklyn, NY 11215 USA

**Keywords:** Trauma Focused Cognitive Behavioral Therapy, Parental incarceration, Children of incarcerated parents, Post Traumatic Stress Disorder, Childhood trauma, Incarceration, Anxiety, Depression

## Abstract

This paper explores children’s trauma symptoms related to parental incarceration and lays the groundwork for the implementation of Trauma Focused Cognitive Behavioral Therapy (TF-CBT) within a clinical community-based setting treating children and adolescents affected by parental incarceration. Children and adolescents who experience parental incarceration are more likely to develop symptoms of post-traumatic stress disorder (PTSD): depression, anger, aggression, and isolating and self-harming behaviors. Although parental incarceration is a known source of trauma, there are no documented studies examining effective clinical treatments to reduce the effects of the trauma experienced by these children and adolescents. Except for children and adolescents affected by parental incarceration, TF-CBT, a promising model for treating and reducing the symptoms of PTSD, has been successfully applied to various populations affected by trauma. Children of incarcerated parents resemble populations treated with TF-CBT in earlier applications. In this paper, we present a case illustration that examines the application of TF-CBT with one child who experienced trauma symptoms related to parental incarceration, while discussing the results of that application and the potential for broader applicability of TF-CBT within community-based organizations that treat the population of children and families affected by parental incarceration.

## Introduction

The United States (U.S.) is the world’s leader in incarceration with 2.2 million people currently in the nation’s prisons and jails, representing a 500% increase over the last 40 years (Glaze and Maruschak 2008). To underscore the extensive nature of this issue in US society, nearly two million children have at least one incarcerated parent, according to the U.S. Bureau of Justice Statistics (Mumola 2000), a number that is likely underreported because it does not consider the custodial states of individuals who are a child’s guardian or who perform other significant caretaking functions (Manning 2011; Miller et al. 2013; Murray and Farrington 2008). Children can exhibit the emotional effects of parental incarceration through complex trauma-related stress symptoms such as isolating themselves from their peers, anxiety, struggling to form healthy interpersonal relationships, concentration problems, sleep difficulties, emotional withdrawal from family members, substance use or dependence, and significant feelings of shame and secrecy. If unaddressed, the impacts of these symptoms can often lead to long-term psychological and emotional functioning problems (Manning 2011; Murray et al. 2012; Miller and Barnes 2015; Phillips et al. 2006). Parental incarceration also threatens the future liberty of the children themselves by placing them at a far greater risk of future criminal justice involvement. According to the U.S. Department of Justice without intervention, 70% of these children will follow in the footsteps of their parent(s) and enter the juvenile and/or adult criminal justice system (Mumola 2000).

Between 1991 and 2007, the number of children with parents in prison has nearly doubled. Nationally within state prisons, approximately 20% of children with an incarcerated parent are less four years old, approximately 60% are between five and 14 years old, and 16% are between 15 and 17 years old. One-third of all children will be 18 years old by the time their incarcerated parent is released from prison (Glaze and Maruschak 2008). For most caretakers, parental incarceration is a negative event—it can produce financial insecurity, elevate emotional stress, strain interpersonal relationships, and increase difficulties associated with the supervision of children (Turanovic et al. 2012). In some uncommon circumstances, parental incarceration can improve the living conditions of a family, specifically in instances where the removal of the incarcerated parent does not negatively affect the quality of the home environment (Turanovic et al. 2012). However, for most family members, parental incarceration has significant negative consequences.

Children of incarcerated parents experience numerous sources of material and emotional insecurity. For example, children of incarcerated parents are more likely to receive public assistance, to experience interrupted phone or utility service due to non-payment, and to experience residential insecurity through missed mortgage and rental payments (Geller et al. 2009). These examples are symptoms of the greater socioeconomic disadvantage that exists for these children even before a parent is incarcerated, and these conditions tend to worsen after incarceration (Miller and Barnes 2015; Phillips et al. 2006). Besides these material consequences, a parent’s incarceration can abruptly dismantle a family. Children of incarcerated parents are less likely to live in a home with both parents present (Geller et al. 2009) and are sometimes placed indefinitely in foster care while family members are sent to distant penitentiaries, making travel and visitation difficult for families with limited resources and few transportation options (Manning 2011).

Using data from Add Health, a national panel dataset that includes 20,700 respondents in grades 7–12, Roettger and Swisher (2011) determined that paternal incarceration is highly and significantly correlated with increased likelihood of arrest before the age of 25 years old. These findings are supported throughout additional studies examining the effects of parental incarceration on a child’s future criminogenic risk. Huebner and Gustafson (2007) found that parental incarceration nearly triples a child’s risk of future criminal justice involvement and that children of incarcerated parents are up to three times more likely to be incarcerated than counterparts who did not experience parental incarceration. A meta-analysis conducted by Murray and Farrington (2008) confirmed these findings and further concluded that these effects were exacerbated when the child was without additional protective factors, like supportive communities, therapeutic outlets, or friends and family they can talk to or if they reside within especially punitive environments. There are significant racial disparities here, as well: in a sample of children who experienced paternal incarceration, black and Hispanic children were respectively 39.2 and 46.7% more likely than white children to be arrested before they were 25 years old (Roettger and Swisher 2011).

Parental incarceration poses an emotional risk on children and, as early as age three, children of incarcerated parents can begin to exhibit signs of emotional distress like aggression and difficulty attaching to primary caregivers (Geller et al. 2009). Well into adolescence and young adulthood, the emotional effects of parental incarceration can be exhibited in the form of trauma-related stress symptoms such as depression, anxiety, difficulty forming relationships, concentration problems, sleep difficulties, emotional withdrawal, substance use or dependence, and significant feelings of shame and stigmatization (Manning 2011; Murray et al. 2012; Miller and Barnes 2015; Phillips et al. 2006). These emotional and behavioral symptoms have far reaching consequences and may partially explain the educational under-attainment and school-based difficulties like truancy, drop-out, and grade failure that are more common among children of incarcerated parents (Murray and Farrington, 2008; Murray et al. 2012; Phillips et al. 2006).

## Children Traumatized by Parental Incarceration and Trauma-Focused Cognitive Behavioral Therapy (TF-CBT)

Arditti’s (2005) ecological approach to incarceration aims to holistically understand the effects of incarceration on the interrelationships between the family members, environments, and society, and asserts that incarceration may produce multiplicative harms on children and families. These interconnected harms—strained parent–child relationships, difficulties associated with traveling to and visiting custodial settings, community reactions to reentry, and stigma—have the potential to reinforce and be linked with one another. Because they experience intersectional sources of trauma, children of incarcerated parents are a highly vulnerable population. However, due to stigmatization and secrecy, children of incarcerated parents are broadly viewed as an “invisible population.” (Phillips and Gates 2011).

Parental incarceration itself can be seen as a symptom of existing trauma and/or a direct cause of trauma-symptom development. The overlapping racial, social, and structural disadvantages experienced by the majority of children of incarcerated parents will make it difficult to isolate the causal effect of parental incarceration on the development of trauma symptoms; even without this information, parental incarceration is a strong predictor of child trauma. Many children affected by parental incarceration experience complex traumas made manifest by sustained and repeated traumatic experiences like racism, poverty, diminished social mobility, lack of access to resources, and low employability that are increasingly common for poor, minority children (Western and Wildeman 2010) and are associated with trauma symptom development (National Research Council 2014). Even prior to their parent’s incarceration, these children experience an elevated risk for trauma-symptom development and social marginalization (Western and Wildeman 2010).

Existing research implies that parental incarceration is associated with numerous negative effects (Manning 2011; Murray et al. 2012; Miller and Barnes 2015; Phillips et al. 2006). Parental incarceration meets the criteria for complex traumatic exposure, which is defined by the National Child Trauma Stress Network Complex Trauma Task Force (2003), as a “[child]’s experience of multiple traumatic events that occur within the caregiving system…” (p. 5). Research conducted by Arditti and Salva (2015) suggests that parental incarceration can significantly predict childhood trauma for children living in single caregiver homes. Manning (2011) describes in vivid detail the ways in which a parent’s arrest and detention has potentially traumatizing effects on the child:

The arrest of a parent is a terrifying experience for a child…[A] large number of officers, dressed in military attire, break down the door and enter brandishing weapons and shouting orders; the parent is handcuffed and taken away. What happens to the children in the home during and after an arrest? Sometimes children are left along to fend for themselves. Sometimes they are put in the back of a police station to await relatives. Sometimes they are taken to juvenile facilities to await a foster placement. So the incarceration cycle begins in a deeply traumatic way: the children are terrorized at the intrusion of heavily armed police officers and watch their parents, taken away, often kicking and screaming. Then they are abandoned either to fend for themselves or to be subjected to an arrest-like procedure (p. 270).

Once a parent becomes involved with the criminal justice system, a child’s exposure to traumatizing events increases. Beginning with the arrest, and continuing through until a parent is released, the risk of exposure to additional traumatic experiences persists. Parental arrest is simply the initiating traumatic event for a child with an incarcerated parent. If the child and their caregiver have the finances to make the expensive trip sometimes hours away, that same child is subject to additional trauma at each subsequent visitation process where he or she experiences the scrutinizing authority of a custodial complex and the indignity of a supervised visit with a parent within a secure setting. As a result of their parent’s arrest and incarceration, these children experience various trauma-related symptoms–depression, attachment issues, emotional withdrawal, sleep disturbances, cognitive delays including attention deficit problems, and relationship issues like difficulty developing trust or establishing autonomy—and these symptoms mirror those typically found in traumatic grief, traumatic loss, and post-traumatic stress disorder (Manning 2011). The presence of secondary traumas (i.e., neglect, poverty, homelessness) may act as mediating variables for trauma symptom development.

TF-CBT has been shown to be quite promising for the treatment of trauma in children who are experience post-traumatic stress disorder and trauma-related symptomatology (Cohen et al. 2015). The aim of TF-CBT is to reduce trauma-related symptomatology in children who experience such symptoms through the provision of individual and family therapy counseling sessions (Cohen et al. 2006). The Substance Abuse and Mental Health Administration lists trauma-focused cognitive behavioral therapy as a therapy “with effective outcomes” for some treatment conditions and as “promising” for all other conditions within its National Registry of Evidence-based Programs and Practices (SAMHSA). In numerous studies, TF-CBT is shown to significantly reduce trauma symptomatology (de Arellano et al. 2014; Konanur et al. 2015). The studied effects of TF-CBT have been favorable. In the 15 randomized controlled trials that evaluated TF-CBT, the treatment group experienced significantly improved symptomatology compared to the control group across outcome measures examining PTSD, depression, anxiety, behavioral, cognitive, relationship and other problems (Jensen et al. 2014; Cohen et al. 2015). In their meta-analysis examining TF-CBT treatment in children and adolescents, de Arellano et al. (2014) concluded that TF-CBT is effective at treating highly vulnerable populations such as children who are at risk for violence, suicidality, psychosis, and/or substance abuse, as well as those who are developmentally disabled. de Arellano et al. (2014) also concluded that TF-CBT is associated with improved client and caregiver outcomes. Individuals experiencing symptoms of post-traumatic stress performed significantly better than individuals in comparison groups. The findings for treatment of individuals with depression symptoms was less robust, however, showing that in 5 out of 9 studies examined, the TF-CBT produced a medium-sized effect compared to individuals not receiving treatment. Findings for individuals experiencing other behavioral problems, such as aggressive behavior, were mixed. The research produced by de Arellano et al. (2014) suggests that the application and usage of TF-CBT is expanding and that even with wider dissemination, fidelity to the model remains strong. This fidelity may suggest some ease in the implication of the model.

A more recent randomized controlled trial conducted by Konanur et al. (2015) found that TF-CBT significantly reduced post-traumatic stress symptomatology during the observed treatment period, suggesting that engagement in this therapeutic process drives positive outcomes. Furthermore, Konanur et al. (2015) provided interesting insight into the specific processes, mechanisms, and contributions of the TF-CBT process. Konanur et al. (2015) were able to hypothesize that positive, non-significant outcomes related to symptom improvement during the assessment period may be related to hope or an optimistic outlook by the client during the introductory period.

TF-CBT also appears to have a greater impact than other similar treatments. For example, in their randomized controlled trial, Cohen et al. (2004) found that children with abuse-related PTSD symptoms and their parents who were treated with TF-CBT experienced more significant improvements than children and families treated with Child Centered Therapy (CCT).

There is limited research, however, on the application of TF-CBT to children who have experienced parental incarceration. A review using the EBSCO database with the search terms “TF-CBT”, “parental incarceration,” and “children of incarcerated parents”, found no studies or clinical reports indicating that this treatment orientation has been applied to children of incarcerated parents. This may be because TF-CBT is still a relatively new intervention and its application to this specific sub-population has not occurred or been documented. The methodological difficulties associated with locating a sample of children traumatized by parental incarceration—an “invisible population”—coupled with issues of secrecy and stigmatization, may also explain the exclusion of this specific group from research examining effective treatment interventions (Manning 2011). There is research suggesting that TF-CBT is an effective intervention for comparable treatment groups, such as children exposed to interpersonal violence and those treated with TF-CBT experienced lower rates of relapse into PTSD symptomatology than the non-treatment group. (Cohen et al. 2011).

Parental incarceration is considered an adverse childhood experience, a designation, which coupled with additional risk factors, has been shown to be predictive of trauma development (Afifi et al. 2011; Arditti and Savla 2015). Many, if not all, children affected by parental incarceration experience the depression, functional and cognitive impairments, anxiety, and disruptive, antisocial, and externalizing behaviors that are each symptoms of trauma. Finally, these risk factors are compounded by a variety of other racial, structural, and environmental risk factors that are likely correlated with parental incarceration, which in and of themselves, expose children to risk for trauma-related symptomology.

Children of incarcerated parents are undoubtedly a high-risk population for trauma-related symptom development and this necessitates the exploration and utilization of effective interventions and treatments that can be adequately applied to this population.

## TF-CBT Background

TF-CBT, a newly developed evidence-based practice, was created and tested by Judith A. Cohen, Anthony P. Mannarino and Esther Deblinger in the late 1990s, and it began to receive significant recognition after therapists applied the model to treat individuals who had been affected by the events of September 11th. TF-CBT aims to reduce distress and resolve maladaptive cognition associated with trauma within a brief treatment period of 12–18 sessions. The first manual was published in 2006 (de Arellano et al. 2014), and the earliest applications of this model to children were by community practitioners who used it to assess and treat traumatic grief in sexually abused children between three and 18 years of age. Due to its versatility, TF-CBT became popular for community-based practitioners working in direct service settings with children and adolescents from diverse cultural backgrounds with challenging clinical presentations and complex family situations (Cohen et al. 2006). With increasing clinical attention given to the emotional distress and complex trauma symptoms experienced by children of incarcerated parents such as difficulty forming attachments, difficulty concentrating and sleeping, inability to develop trust, and achieving identity (Manning 2011), this model presently offers a new option to practitioners who work specifically with this population.

TF-CBT, like all manualized treatments, adheres to a precise treatment model. To ensure that children screened to receive treatment are an appropriate fit, the therapist must possess a total comprehension of traumatic events. The core components of TF-CBT are psychoeducation about the child’s identified trauma and PTSD symptoms; affective modulation skills; individualized stress-management skills; an introduction to the cognitive triad (relationships between thoughts, feeling and behaviors); creating a trauma narrative (a gradual exposure intervention where the child describes the progressively distressing details of their trauma); cognitive processing; safety skills and education about healthy healing; and a parental treatment component (Cohen et al. 2004). TF-CBT provides an intervention by which a child can address the stigmatization they experience as children of incarcerated parents, and offers a clinical approach to help them heal from emotional, physical, and material consequences resulting from the traumatic loss of an incarcerated parent. Grounded in the principles of cognitive-behavioral therapy, TF-CBT likewise emphasizes the importance of the patient-caregiver relationship to help develop the child’s emotional regulation and coping capabilities (de Arellano et al. 2014).

TF-CBT requires parental involvement in treatment through the joint treatment sessions with the child and caregiver or guardian. These cooperative sessions aim to provide caregivers with skills that allow them to expand learning from psychotherapy into the child’s home (Konanur et al. 2015). As the family acquires a more comprehensive understanding of the child’s symptomatology and experience, the goal of reducing the child’s traumatic symptoms can be more readily achieved. When implementing TF-CBT, therapists are encouraged to adapt the model to fit the needs of the children and family members and to facilitate enhanced cognition and understanding of trauma in the caregiver. The therapists speak directly about the trauma as it is crucial in resolving and understanding the obstacles and integrating the experience into the child’s life in an optimal way. This happens progressively and through partnership with the parent throughout treatment (Cohen et al. 2006). By naming the child’s trauma, they then identify what they would like to focus on for treatment. The case example to be discussed highlights how the client self-reported her mother’s arrest and incarceration as being her most painful trauma as it is suggested in this model that the child guide the therapist to which traumatic experience should be included for the narrative (Cohen et al. 2006). Because people of varying religions, technicalities, and cultures have diverse ways of expressing and coping with traumatic responses and reactions, the therapist must view the child and parent as the experts and learn from them what their rituals, beliefs, and practices are within their culture, family, and individually. This is to always to remain respectful throughout the entire TF-CBT treatment process (Cohen et al. 2006). Also, given the stated effects of parental incarceration on the remaining caregivers, its crucial to point out that the family treatment approach that is a component to TF-CBT provides opportunities for treatment of the strained caregiver responsible for the child.

When employed within the agency setting, this model will allow for the children and parent/guardian to quickly engage in a process that safely explores the language and definitions of trauma that begin the healing process necessary to restore the child’s ability to function on a day-to-day basis both at home and at school. The core components used within the model frame the clinical work to empower the child and caregiver to simultaneously learn about their trauma symptoms while effectively master the coping mechanisms necessary to manage overwhelming feelings.

## Case Example of TF-CBT Treatment for Children Traumatized by Parental Incarceration

The following case study is an example of a local deployment of TF-CBT treatment to a child and caregiver dyad within a community-based outpatient setting. This case study discusses the associations between parental incarceration and the development of trauma-related symptomatology, the treatment conditions addressed through the TF-CBT intervention, initial outcomes from treatment, as well as the implications for TF-CBT utility for children affected by parental incarceration. In this description of their treatment, all names and identifying information have been altered to protect patient confidentiality. Signed informed consent for publishing this case example was obtained by the caregiver and the child assented.

### Presenting Problem: Case Conceptualization

Serena, a 23-year-old African American woman sought mental health services for her 9-year-old daughter, Lelani. During an initial telephone screening with a supervising clinician, Serena explained that she was incarcerated at a New York State correctional facility and had been referred to the clinic because of its mission to work with children and families affected by incarceration. Serena provided an initial history and background information for her family. Serena had been physically and sexually assaulted on numerous occasions over the course of 10 years by her daughter’s father, Terrence.

On the night of the arrest that led to her incarceration, Serena stabbed Terrence in self-defense during a brutal physical attack that included an attempted rape. Lelani awoke as the paramedics arrived to remove her father and observed the police handcuff and remove her mother. Lelani describes crying for her mother as several officers forced distance between them. Unable to approach her mother, she screamed out that she wanted to “go with mommy”.

While her mother was detained, Lelani was placed in kinship care with her maternal grandmother. Serena reported that Lelani had become emotionally withdrawn in the immediate period following her arrest and detention and began to exhibit certain behavioral problems at school. Serena shared that her mother would call her and report that Lelani consistently stated she wanted to “see mommy” and “be with mommy”. Serena admitted that she was still struggling with the emotional effects of the sustained assault and victimization and had been recently diagnosed with complex PTSD while she was incarcerated.

Serena received a conditional, early release from prison and returned home. She resumed care of Lelani, still under the kinship care with her maternal grandmother, and quickly decided to meet with social workers for an in-person intake and assessment. Social workers conducted an initial screening with both Serena and Lelani present. The initial intake was an opportunity for social workers to help Serena and Lelani discuss their feelings about the incarceration and to identify the residual clinical implications of these events. During the first section of the intake, social workers met with the child and caregiver together. Serena explained that Lelani had acted aggressively on multiple occasions in school, which resulted in numerous suspensions. During this initial intake, Serena revealed that she had felt “afraid” for her daughter and “ashamed” while she was incarcerated. Serena presented herself as someone with low self-esteem, driven partially by the hopelessness she felt during her imprisonment. “I couldn’t help her,” she told the social workers. As part of the screening process, social workers directly engaged Lelani in a discussion around the altercation that resulted in Serena’s arrest and attempted to identify her thoughts and feelings surrounding the traumatic event and the subsequent years of her mother’s imprisonment.

### Application of TF-CBT with Serena and Lelani

Over two ninety-minute intake sessions, the clinical team administered a comprehensive biopsychosocial assessment, trauma assessment and checklist, PTSD-Reaction Index, adult trauma checklist, and psychiatric evaluations to identify any ongoing chronic traumatic stress and symptoms experienced by both Serena and Lelani. A clinical treatment plan was established for both clients, which recommended that Serena and Lelani would each be seen once weekly for individual psychotherapy. The treatment plan also recommended that the family receive joint sessions on a weekly basis over the course of 18 weeks and Lelani requested to participate in group therapy that was offered onsite for children impacted by parental incarceration. During this intake process, the social workers explained to the family that they would be treating them using TF-CBT with three phases of the model. The model was described in further detail to the family as consisting of the following three phases: (1) the stabilization phase, (2) the trauma narrative phase, and (3) the integration/consolidation phase. A brief description of the activities and purposes of each phase was also described during this assessment.

#### Phase 1—Stabilization

During the stabilization phase of the model, social workers used psychoeducation to discuss trauma and domestic violence with Serena and Lelani within sessions one to three. The social workers determined that psychoeducation around these subjects was necessary because Lelani commonly expressed feeling as though “daddy beat up mommy because she was bad” which encouraged the exploration of what domestic violence is and how her mother had been a victim of abuse. When describing instances of her own abuse, Serena shared that she “just thought bad things always happened” showing evidence of dysfunctional cognitions (Unterhitzenberger and Rosner 2016).

Throughout sessions four to seven of both individual psychotherapy and joint sessions during this phase, Lelani reported that many children at school would ridicule her for being parentless throughout the period of her mother’s incarceration. Community members called Serena a “monster” and “murderer” in front of Lelani who lashed out at her mother for “taking away [her] daddy.” In individual psychotherapy sessions with the caregiver during this phase, social workers helped Serena connect her daughter’s behavioral problems to the traumatic experiences being discussed in their therapeutic process. The social worker aided Serena in embracing a realistic perspective of the reason for her daughter’s behavior through employing the Cognitive Triangle, commonly used as a therapeutic tool throughout TF-CBT, to accurately identify her feelings and better understand how her thoughts and feelings impacted her reactions towards her daughter. As a result, Serena was able to regulate her reactive responses to her daughter’s insults through the application of parenting exercises where she learned how to provide Lelani with praise and reinforcement, as well as selective attention when she would be negatively acting out. This allowed Lelani to have room to safely express herself without Serena becoming reactive.

Lelani responded strongly to the mindfulness practices and focused breathing techniques taught during sessions eight to nine of this phase. She explained that when provoked she would “fight anyone who got near her” and her grandmother revealed that she had been suspended several times during her mother’s incarceration and was unable to connect to any adult figures because she feared they would leave her. The social worker aided Lelani in understanding the physiology of relaxation and how she could successfully regain control over her body when she felt angry or upset. When she began to effectively implement these relaxation and mindfulness practices, such as deep breathing and positive self-talk at school, the family was overjoyed that she was reportedly no longer engaging in physical altercations. The social workers taught relaxation through guided meditation and affective regulation skills, which enabled Serena to recount the abuse she endured. She was also able to recall how she would go into fits of rage, or “black outs”, that she would not remember. Through cognitive processing, an integral element of the stabilization phase, the social workers explained to Serena that these “black outs”, were in fact symptoms of trauma caused by abuse and “torture” as described by Serena during her own individual psychotherapy sessions. The stabilization phase of the model also helped both participants prepare for the narrative writing that would be executed in the next phase of the model.

#### Phase 2—Trauma Narrative

Completion of the first phase of the TF-CBT model facilitated Lelani’s understanding of the abuse her mother endured and that the act that led to her incarceration was carried out in self-defense. Still, she requested to write about her mother’s arrest and incarceration. When it came time for Lelani to write and deliver her trauma narrative to Serena, she chose to focus her story on her mother’s arrest and what she experienced while she was separated from her mother. In sessions 10–12 Lelani developed her first draft of the narrative. During the formulation of the narrative, Lelani developed an interest in better understanding the events surrounding the night of her mother’s arrest and expressed a desire to ask her mother questions about the events in their joint family sessions. Lelani shared feeling “not as angry” and “just sad because I lost my mommy for two years and my daddy for good all in one night.” She wanted to know if she “would ever get another daddy again” and if she would “lose mommy again to the police”. Lelani included these feelings in the narrative and had an emotional and powerful reading of the story in session 13. This was the first time her mother heard from Lelani that she did not blame her mother for protecting herself, but that she needed to share her feelings of pain and sadness for no longer having her father. Lelani was able to vocalize her feelings and Serena was willing to listen without reacting in a negative manner. Through the reading of the narrative Lelani no longer covered her face when showing avoidance, she was able to confront her fears and speak directly to her mother. Lelani said she learned to do this by “deep breathing” instead of hiding her face.

#### Phase 3—Integration/Consolidation

During sessions 14–17, the final phase of treatment, the social workers continued co-joint parent–child sessions. Lelani and Serena enhanced their overall communication and developed and practiced a safety plan to address her social and emotional behaviors (e.g., counting to 10, closing her eyes while practicing deep breathing, conflict mediation). Lelani and Serena spoke openly about the trauma they experienced during Serena’s incarceration and the death of Lelani’s father. Serena expressed feeling “hopeful about their growing relationship.” Because she was able to address the overwhelming shame and guilt engendered during her imprisonment, Serena’s self-esteem and self-efficacy improved dramatically throughout the course of treatment through self-reports in both individual and family therapy. The narrative sharing process facilitated the development of trust between the child and caregiver. Although she initially was afraid to express her desire for her mother’s protection from the community’s shame and stigmatization, the narrative process enabled Lelani to share her true feelings with her mother and “feel safe to share everything with my mommy”. In their last session, they reported that they had a plan to “move forward and grow as a family”.

### Case Discussion

As illustrated in this case study, parental arrest and incarceration may inflict multiple traumatic events on a child that occur along a sequalae of events. Trauma may occur prior to a discrete event that results in arrest, during the actual event or arrest, and/or after a parent has been incarcerated. Prior to arrest, the child may have experienced a variety of structural, emotional, and psychological traumas, including: structural disadvantages related to racism or poverty; exposure to unsafe residential conditions, residential instability, or financial insecurity. During and immediately following the precipitating event, the child may have experienced other traumas, including: direct observation or experience with law enforcement officials related to the arrest; and direct observation of a parent’s removal by law enforcement officials. Lastly, over the long-term period between a parent's arrest and continuing until and after a parent’s release, the child may have experienced even more additional traumas, including: limited exposure to their parent during detention and any subsequent period of incarceration; direct experience with custodial officers at detention facilities; persistent or sustained residential or financial insecurity as a result of the arrest, including placement in foster care settings; increased risk for his or her own criminal justice system involvement; increased likelihood of reduced achievement in school settings; stigma associated with parental incarceration; and/or unresolved loss.

This case carefully demonstrates that the acute and chronic events leading up to and ending in parental arrest can be in and of themselves traumatic for a child. Lelani was exposed to the loss of both parents in one night, an event that resulted in her mother’s arrest and subsequent incarceration, which was then succeeded by her experience of a variety of long-term traumas resulting from her parent’s incarceration. Lelani ultimately presented with a variety of risk factors for trauma-exposure and was treated with TF-CBT because of its individual and family therapeutic components and its promise for reducing trauma-related symptomatology. During her treatment, Lelani and her therapist focused on the many traumatic events she experienced in her life that lead to, and ultimately, continued up until she and her mother sought clinical services. Throughout the course of treatment, Lelani worked to untangle the loss she experienced with her mother’s incarceration and this work continued into long-term care post completion of the model that aimed to address the violence that resulted in the murder of her father. After completion of the TF-CBT model, to deepen their treatment, both Lelani and Serena continued to utilize long-term ongoing psychodynamic therapy and Lelani continued to participate in group therapy. In her long-term care, the clinical team worked with Lelani to uncover the complex and difficult feelings she felt towards her mother for the events that resulted in her father’s death. In Serena’s long-term care she was also able to address the domestic violence she survived, her experiences while incarcerated, and the changes and difficulties that Lelani displayed in the period following her mother’s incarceration.

This case underlines the utility and promise of TF-CBT treatment for children who are experiencing parental incarceration. While parental incarceration is likely one of several risk factors associated with the development of complex trauma, traumatic grief and loss, and PTSD, the existence of the condition of parental incarceration, coupled with numerous other high-risk factors suggests that the children could benefit greatly from this model as a form of intervention throughout long-term care and support.

This case demonstrates that TF-CBT can be effectively used with a child and parent suffering from traumatic symptoms related to parental incarceration and other complex traumas. The case illustrates how the core concepts of this model allowed for the family to speak openly and safely without feeling afraid that any member would become overwhelmed or upset when speaking about the traumatic separation during the incarceration. This particular illustration underlines the importance for both child and caregiver of regular participation in their own individual therapy because these individual sessions provide space for each client to share their concerns before the joint sessions without causing more distress.

The description of the phases throughout the case example convey that no significant variations were made to the model by the social workers. To maintain cultural competency and remain client-focused, the social workers and family members ensured that Lelani identify the trauma most important to her to be used for the narrative. One of several goals for developing the trauma narrative is to “unpair thoughts, reminders, or discussions of the traumatic event from overwhelming negative emotions such as terror, horror, extreme helplessness, shame, or rage” (Cohen et al. 2006, p. 119) over the timeline of multiple sessions. Therefore, the therapists supported Lelani in the time she needed to write and eventually read the narrative to her mother once she felt emotionally safe and prepared. This practice would be used with any client throughout the course of treatment and does not deviate from the guidelines of TF-CBT (Cohen et al. 2006). Using the psychoeducation offered throughout the model, Serena was able to comprehend a more accurate depiction of her years of abuse and what essentially lead to her having to defend herself that night. This allowed Serena to feel relieved of the guilt and excessive worry she felt when Lelani would become angry at her during an aggressive episode at home, and Serena became able to successfully respond with patience. When the model concluded, the family revealed feeling “lighter” and “closer” than they had prior to her Serena’s incarceration. Both Serena and Lelani verbally reported feeling that their symptomatology had improved and that they had attained the goals established in their treatment plans.

## Limitations of TF-CBT

TF-CBT applies a prescriptive approach according to a specific model of treatment. Research conducted by Cohen et al. (2006) on treatment resistance has suggested that social workers themselves may experience resistance to implementation of evidence-based treatments because they may perceive manualized models to be more rigid and less creative than other therapeutic approaches to treatment. Because TF-CBT is a short-term model, social workers may also feel as though they are pressured by a time constraint that favors the completion of task-oriented client experiences and activities, as opposed to carrying out ad hoc clinical procedures. Social workers may also experience their own avoidance when discussing trauma, which can hinder the clinical process further (Simonich et al. 2015).

While the model works most efficiently for children who present higher rates of depressive symptoms and have a willingness to engage in the therapeutic process, clients who are experiencing stronger symptoms of trauma avoidance and mistrust in authoritative figures may struggle to initially connect to their therapists. This can cause critical delays in the application of this time-specific framework. In addition, one study found that symptom arousal and clinical withdrawal increased during the assessment period, suggesting that service initiation may increase symptom intensity especially in cases where avoidance is common and where children may have difficulty tolerating these arousal symptoms early in the therapeutic process (Konanur et al. 2015).

Documented research suggests that TF-CBT is efficacious when employed with fidelity to the model, even when applied to other populations. Furthermore, most research about TF-CBT is generally limited to a few studied outcome measures, such as reduction in symptomatology of depression and PTSD, improvement in general and sexual behaviors, and improvement in parenting behaviors for non-offending caregivers (de Arellano et al. 2014; Brady et al. 2015). Another explanation for limited expansion of treatment application may be related to the limited resources that are available within the community-based settings carrying out the efficacy trials; without necessary staff and resource support, programs may struggle to implement and properly evaluate this intervention (Konanur et al. 2015).

TF-CBT is by no means the sole treatment option available for individuals effected by PTSD or trauma-related symptoms. For example, in their meta-analysis exploring the effectiveness of a variety of psychotherapeutic treatments, Bradley et al. (2005) concluded that “a variety of treatments, primarily exposure, other cognitive behavioral therapy approaches, and eye movement desensitization and reprocessing are highly efficacious in reducing PTSD symptoms.” (p. 225) TF-CBT should be understood as a promising treatment that should be utilized when its prerequisite conditions for treatment can be met. For example, because TF-CBT includes a substantial family therapy component, if a clinician is treating a client without strong family ties, it might be more appropriate to exercise an alternative approach, of which there are many. In addition, many individuals experiencing PTSD or trauma-related symptoms require additional follow-up even after the initial treatment regimen has concluded. For these reasons, clinicians who deploy TF-CBT should monitor their clients during the follow-up period and continue treatment with other suitable modalities as needed. (Bradley et al. 2005).

## Conclusion

Children of incarcerated parents, marginal as they may be, are not a nominal population and additional research into TF-CBT success with this population is further justified by the innate vulnerability of the population. At least 10% of all Black children under the age of ten had one parent in prison or jail in 2000. (Western and Wildeman 2009). It cannot be overstated that, alongside parental incarceration, the mediating effects of racism, poverty, low educational attainment, and a variety of other social barriers make it essential for the social worker to acknowledge that a client’s socioeconomic and demographic circumstances can interact with or contribute to their trauma (Geller et al. 2009). Given this disturbing association between incarceration and race, clinicians need to be especially aware of issues around cultural sensitivity and should acknowledge and respond to these differences to maximize therapeutic input (Meyer and Zane 2013). Success of TF-CBT requires the development of a therapeutic alliance, which will only be effectively created if the therapist is sensitive to the cultural differences of the clientele. As was critical in the case of Lelani and Serena, the TF-CBT treatment must be performed by a clinician who is capable of recognizing the disproportionate impact that incarceration has had on impoverished persons of color, while simultaneously acknowledging and being prepared to discuss cultural further differences in the interest of normalizing the client experience.

A case example provides only limited evidence for the treatment model’s success and additional research must be conducted to develop more conclusive evidence that TF-CBT is an effective treatment model for this population. Some research suggests that the trauma caused by parental incarceration can be mitigated by increasing the frequency of parental visits. (Arditti and Savla 2015). However, even if the condition of parental incarceration is ameliorated, it is unclear if other mitigating environmental factors that affect these populations (e.g., poverty, crime, unemployment, etc.) would prevent or reduce the potential for future success and reduction of symptomatology.

TF-CBT is evidence-based to reduce the severity of post-traumatic symptomatology in treated clients; however, given the nature and intensity of a client’s presenting symptoms, sustained participation in some additional clinical treatment may be necessary and beneficial. The results presented through this case example should encourage future researchers to explore this treatment intervention with a larger sample in order to produce results that are more generalizable and conclusive. Through further dissemination of the therapeutic model and examination of additional treatment outcomes TF-CBT’s efficacy with this population may be better determined.
